# Domestic Dogs Use Contextual Information and Tone of Voice when following a Human Pointing Gesture

**DOI:** 10.1371/journal.pone.0021676

**Published:** 2011-07-13

**Authors:** Linda Scheider, Susanne Grassmann, Juliane Kaminski, Michael Tomasello

**Affiliations:** 1 Department of Developmental and Comparative Psychology, Max-Planck Institute for Evolutionary Anthropology, Leipzig, Germany; 2 Department of Developmental Psychology, University of Groningen, Groningen, The Netherlands; Università di Parma, Italy

## Abstract

Domestic dogs are skillful at using the human pointing gesture. In this study we investigated whether dogs take contextual information into account when following pointing gestures, specifically, whether they follow human pointing gestures more readily in the context in which food has been found previously. Also varied was the human's tone of voice as either imperative or informative. Dogs were more sustained in their searching behavior in the ‘context’ condition as opposed to the ‘no context’ condition, suggesting that they do not simply follow a pointing gesture blindly but use previously acquired contextual information to inform their interpretation of that pointing gesture.

Dogs also showed more sustained searching behavior when there was pointing than when there was not, suggesting that they expect to find a referent when they see a human point. Finally, dogs searched more in high-pitched informative trials as opposed to the low-pitched imperative trials, whereas in the latter dogs seemed more inclined to respond by sitting. These findings suggest that a dog's response to a pointing gesture is flexible and depends on the context as well as the human's tone of voice.

## Introduction

Domestic dogs (Canis familiaris) are very skillful in understanding some forms of human communication, in particular, the pointing gesture [1]–[4]. Experimental studies have mainly investigated dogs' comprehension of pointing gestures in the so-called object choice paradigm. In this paradigm a reward (e.g., food) is hidden in one of usually two identical cups and the experimenter provides the dog with a communicative cue, e.g., by pointing (and/or gazing) at the correct cup. The dogs are then free to make a choice between the potential locations of the hidden food. A growing body of research demonstrates that, compared to other non-human species, dogs are highly skilled with these forms of communicative signals from humans [2], [5]–[7].

All previous studies investigating dogs' comprehension of the pointing gesture have used some type of reward (e.g., food) as a motivational device to get the dogs participating. Frequently the reward will serve as the object-referent of the human's communicative gesture. As a consequence, in these studies, dogs have always been exposed to reward-related situations. Thus we only know about dogs' comprehension of the pointing gesture in situations where dogs are highly motivated to find something like food. And indeed in this context even a gaze cue without pointing is sufficient to enable dogs to locate the hidden food [2].

One exception is Agnetta et al. who used a non food-related task [8]. Agnetta et al. tested dogs in a gaze-following task where a human experimenter attempted to direct the dog's gaze to one of three predetermined locations (straight up, directly to the left, or directly to the right of the dog) by turning her head and looking at that location for approximately 5 seconds. No reward was provided for any particular response. A response was measured as looking at the three possible target locations or elsewhere (e.g. experimenter). The results showed that dogs do not reliably follow human gaze in such non-foraging situations. In a similar study, investigating their level of comprehension of a human's directional gaze and head nodding cues, Soproni et al. (2001) found that dogs did not follow a human's gaze direction to an empty location above a target object, as opposed to indicating the object directly. Their study was conducted using a two-way food choice task. Therefore food was present as a motivational device and dogs had to choose between two containers. But with no referential component (target object) in the gesture dogs were not able or not motivated to follow it.

Taken together, these results suggest that dogs need the accompanying referential component (object referent) to fully comprehend the communicative intention behind a human's gaze cue. Thus, for dogs it seems that communication needs to be about a referent. And the mere presence of food in the communicative situation does not seem to change this finding. This suggests that gazing is not a cue that is used by dogs simply because it is based on an association with the presence of food. Both of the aforementioned studies, however, investigated dogs' understanding of a human's attentional state that is directed to different target directions. They did not specifically investigate dogs' comprehension of human cues that are intentionally communicative.

To our knowledge no study has investigated how dogs would respond to a pointing gesture with no referent but which is clearly meant as a communicative act.

Dogs' behavior in this kind of situation would provide valuable information about the mechanisms which underlie their comprehension of a human's pointing gesture. If dogs follow this gesture regardless of contextual or referential information in a communicative situation one would be inclined to regard this more as associative behaviour. In other words, the human's hand is associated with food and therefore dogs follow that hand direction regardless of contextual and/or referential information. Also, dogs may interpret pointing as a command ordering them to move to a certain location or in a particular direction, irrespective of the context established [9].

In the current study, therefore, we addressed the question of how dogs would respond to a pointing gesture with accompanying gaze-alternation to an empty location.

We used a 2×2×2 design. In a between-subjects factor, the experimenter pointed to an empty spot on the ground versus no gesture was used whatsoever (control).

In a second between-subjects factor, for some dogs we established a food-searching context while for others no such context was established (no context). From the previous studies on gaze-following, we expected dogs to regard a context in which food had previously been discovered to be more relevant than one in which no food had ever been present. The other question of the study was whether dogs would differentiate between the experimenter's tone of voice, which was varied within-subjects to be either informative or imperative – to see whether the human's vocally expressed motive to order the dog to do something versus to inform the dog of some information would have an effect.

## Methods

The presented study is not invasive. IRB approval was not necessary for this kind of study because no special permission for use of animals (dogs) in such socio-cognitive studies is required in Germany. All procedures were performed in full accordance with German legal regulations and the guidelines for the treatments of animals in behavioral research and teaching of the Association for the Study of Animal Behavior (ASAB). All dogs were registered in the dog database of the Department of Developmental and Comparative Psychology (MPI EVA) and were recruited by phone. All dog owners with their dogs participated on a volunteer basis.

### Subjects

Forty-eight dogs (25 females, 23 males) of various breeds and ages (M = 4,7 years; age range: 1–12,5 years) participated in this study and were included in the analysis. All subjects lived as pets with their owners and were tested at the Max-Planck Institute for Evolutionary Anthropology in Leipzig, Germany.

All dogs had received the training typical of pet dogs. The owners were registered on a database at the MPI EVA and had agreed for their dogs to participate in the study.

The pre-conditions for participation were that the dogs had to be food motivated and comfortable remaining in a testing room without their owners. Three dogs were excluded from the study prior to testing because of anxiety in the testing room. The study was conducted in quiet rooms at the MPI EVA (3,6 m×2,9 m). Recordings were made with one camera (Panasonic NV-GS180) fixed to the ceiling and the room was filmed from above using a special wide-angle lens (“fish eye”, Sony Sakar, 37 mm; 0,45x).

### Experimental Design

Presence versus absence of a food searching context and the absence and presence of a gesture was varied between subjects.

Dogs were grouped such that one group received the experimental condition (with pointing gesture), while the other group received the control condition (no pointing). In those groups subjects were again grouped such that one group received the context trials (food present), while the other group received the no-context trials (food absent).

The experimenter's motive (imperative vs. informative) was presented as a within subjects factor. This resulted in a 2×2×2 design with the following 4 experimental conditions: experimental-context-informative, experimental-context-imperative, experimental-no context-informative, experimental-no context-imperative and the following 4 control conditions: control-context-informative, control-context-imperative, control-no context-informative, experimental-no context-imperative.

Each dog received 8 trials in total. In half of those trials the experimenter's communicative motive was informative while in the other half it was imperative. The informative and imperative trials were blocked such that half of the dogs in each group started with the informative trials followed by the imperative trials, and vice versa for the remaining dogs in that group. The position of the experimenter and the location the experimenter pointed to was counterbalanced across trials and semi-randomized, with the stipulation that the experimenter should never be in the same position and should never point to the same location in more than two consecutive trials. When the experimenter pointed to a location on her right she gestured with her right arm and respectively for the left. Dogs were allowed to move freely throughout the duration of the trial.

### Procedure

#### Pre-Phase

**Figure 1:**
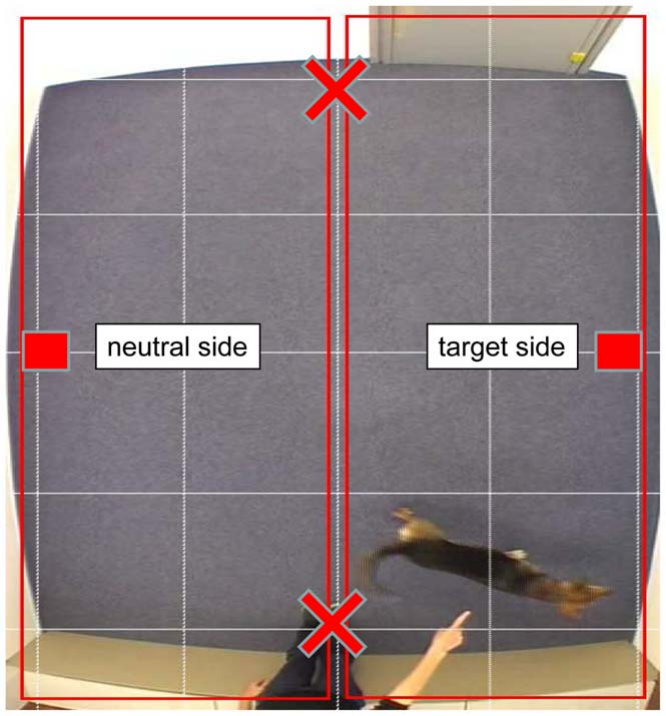
Experimental set up. The room is digitally divided into two parts (“target side” and “neutral side”). The two crosses showing the possible positions of the experimenter (and at the same time the locations where the food was placed in the pre-phase). The boxes show the potential (empty) target locations that the human could have been pointing at.

To establish the context of the respective group of dogs, the dog participated in a pre-phase. The procedure was as follows. First the experimenter guided the dog by its collar into a waiting room adjacent to the testing room. During the time the dog spent alone in the waiting room the experimenter entered the testing room with a piece of food grasped with tongs to prevent her hands from smelling of food. She placed the food on the ground in a predetermined location (see fig. 1) and left it there in the context trials, but removed it after a few seconds in the no context trials. The rational behind this was to control for odor. The experimenter then guided the dog by its collar from the waiting room into the testing room. The dog was allowed to move freely while the experimenter walked once around the testing room without paying the dog any attention. This was to introduce dogs to the food-related situation. The pre-phase lasted approximately 30 seconds.

In the context trials every dog found the piece of food without the experimenter needing to indicate it in any way and the dogs were free to take it without the experimenter attending to their behavior. The latency of finding and eating the food was approximately 4 seconds.

The no context trials were identical to the context trials with the exception that the dog found no food. The rational behind this was to keep the procedures of both groups comparable.

After a short inspection of the room by both the experimenter and the dog, which lasted approximately 10 seconds, both left the testing room and waited outside in the hallway for one minute. After the waiting time had elapsed the dog and experimenter proceeded to the experimental phase of the trial. This procedure was the same in both the experimental and the control condition.

#### Experimental trial

After the pre-phase the dog and the experimenter reentered the testing room. The experimenter guided the dog by its collar into the testing room and then the dog was free to move. The experimenter stood on the location where the food had been placed beforehand. This was done to prevent dogs coming to that spot. They could potentially have smelled where the food had previously been placed, they may have been drawn to it, and this could have caused a side bias. The experimenter stood on the wall facing the room. She waited two seconds, called the dog's name and then pointed at a predetermined location (Figure 1).

During pointing she altered her gaze between the dog and the target location three times and simultaneously said “da!” (German equivalent for “there!”) either in a high-pitched, friendly voice (informative trials), or with a strong a command-like voice (imperative trials) (Movie S1). When it was time for the experimenter to stop gaze alternating she maintained her gesture and gaze directed at the target location for ten seconds. After the 10 seconds had elapsed she switched her body posture, looking up at the ceiling with her arms hanging beside her body. This lasted 30 seconds and then the trial ended. The next experimental trial started with the pre phase of that trial.

#### Control trial

The procedure was exactly the same as in the experimental trials except that the experimenter did not point for the dogs at any time. Instead of pointing the experimenter changed her body posture after addressing the dog and alternating her gaze in the very same way as described in the experimental trials. While alternating her gaze she addressed the dog in the very same way as described in the experimental trial. She looked up at the ceiling with her arms hanging besides her body. This lasted 40 seconds and then the trial ended. The rational for the control condition (additional to the randomized positions of the experimenter and target locations) was to investigate whether the pointing gesture is the main reason for dogs to decide for one side of the room over the other.

#### Scoring

Before analyzing the videos a grid was superimposed over the footage using the program Adobe Premiere (version 2.0). The grid divided the testing room into two halves to measure the dog's location in the room. As for behavioral measurements we observed two main actions. ‘Search’ was defined as when the dog directs its nose to ground and the dog's head is lowered. ‘Obedience’ including ‘sit’ and ‘lay’ (‘sit’ was defined as every position where the dog's hind legs rested on the ground keeping its forelegs straight and ‘lay’ was defined as every position where all four of the dog's legs and belly rested on the ground). In the experimental condition the experimenter always pointed to a predetermined spot in the room (Figure 1). The side to which the experimenter pointed was termed the “target side” and the other half of the room was termed the “neutral side”.

In the control condition positions were the same except the experimenter did not use a pointing gesture. For statistical analyses the dependent measures were 1) the frequency and duration of the dogs' searching and 2) the frequency and duration of the dogs' obedience during the first 10 seconds after the experimenter started addressed the dog. The videos were then analyzed using the program Interact (Mangold, version 9.1.0).

A visual inspection of a plot of residuals against predicted values showed no pattern; we therefore concluded that an ANOVA can be conducted. A second coder coded 20 percent of the original video material with Interact for reliability purposes.

Reliability was good for the behavioral measurement ‘obedience’ (duration: Pearson r = 0.915; frequency: Pearson r = 0.899) and good for the measurement ‘search’ (duration: Pearson r = 0.855; frequency: Pearson r = 0.865).

## Results

We began looking at the duration and frequency of the dog's searching behavior in the experimental and the control condition (between subject factor: condition) as well as the context, the no context conditions (between subject factor variant) and in the informative and imperative trials (within subject factor intonation) regardless of the two halves in the room. Therefore two 2 (condition)×2 (variant)×2 (intonation) repeated measures ANOVAs were conducted. Neither for the duration nor for the frequency measurements there was a significant interaction between the three factors condition, variant and intonation (duration: F(1,44) = 2.042, p = 0.160; frequency: F(1,44) = 0.340, p = 0.563). Additionally there was no interaction between the factors intonation and variant (duration: F(1,44) = 1.776, p = 0.189; frequency: F(1,44) = 0.340, p = 0.563) but there was a significant interaction for the frequency measurement (F(1,44) = 6.753, p = 0.013) and a trend for a significant interaction between the factors intonation and condition for the duration measurement (F(1,44) = 3.812, p = 0.057) indicating that dogs searched longer and more often in the informative trials over the imperative trials but only in the experimental condition, not in the control condition. Furthermore there was a significant interaction between the factors condition and variant showing a longer searching behavior in the context trials over the no context trials but only in the experimental condition (F(1,44) = 5.087, p = 0.029) and only for the duration not for the frequency measurement.

There was no main effect of the factor intonation (duration: F(1,44) = 0.179, p = 0.675; frequency: F(1,44) = 2.365, p = 0.131) but there was a main effect of the factor variant for the frequency measurement showing that dogs searched more often in the context trials over the no context trials (F(1,44) = 30.465, p<0.001) across conditions.

For the frequency measurement there was a main effect of condition (F(1,44) = 7.794, p = 0.008) but not for the duration measurement (F(1,44) = 3.297, p = 0.076) indicating that dogs searched more often but not longer in the experimental over the control condition.

We then looked at the same measurements with respect to the room divisions (target side vs. neutral side). Two 2 (condition: experimental vs. control)×2 (variant: context vs. no context)×2 (intonation: informative vs. imperative)×2 (halves: target vs. neutral) repeated measures ANOVAs indicated that for both, duration and frequency measurement there were neither four-way nor three-way interactions between any factors.

**Figure 2:**
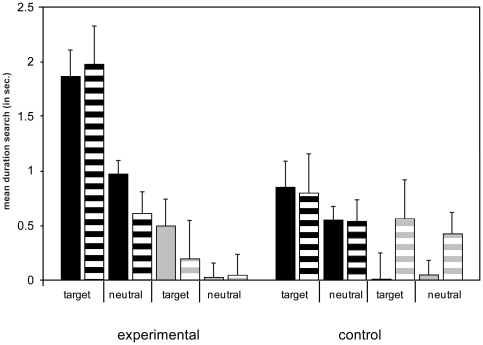
Searching behavior. Mean duration (in seconds) of the searching behavior in “target side” and “neutral side” in the experimental and the control condition, the context and no context trials and the informative and imperative trials (SE). Black bars represent the context trials; grey bars represent no context trials. Non-lined bars represent informative trials, lined bars represent imperative trials.

However, there were the following two-way interactions. For the duration measurement there was a trend for a significant interaction and for the frequency measurement there was a significant interaction between the factors intonation and condition showing again that dogs searched more often and longer in the informative trials over the imperative trials but only in the experimental condition (duration: F(1,44) = 3.812, p = 0.057; frequency: F(1,44) = 7.958, p = 0.007). Additionally, an interaction between the factors halves and variant only for the duration measurement revealed that dogs searched longer in the target side over the neutral side but only in the context trials (duration: F(1,44) = 4.588, p = 0.038; frequency: F(1,44) = 3.174, p = 0.082) (see fig. 2). An interaction in each ANOVA both between the factors halves and condition revealed that dogs searched longer and more often in target side over neutral side but only in the experimental condition (duration: F(1,44) = 5.224, p = 0.027; frequency: F(1,44) = 11.450, p = 0.002). Another interaction between the factors condition and variant revealed again that dogs were searching longer and more often in the context trials over the no context trials but only in the experimental condition (duration: F(1,44) = 5.087, p = 0.029; frequency: F(1,44) = 4.725, p = 0.035) (Figure 2).

Furthermore it was revealed that there were main effects of halves for search behavior in duration (F(1,44) = 13,396, p = 0.001) and frequency (F(1,44) = 12,994, p = 0.001) as well as for the factor variant (duration: F(1,44) = 23.141, p<0.001; frequency: F(1,44) = 31.730, p<0.001). For the frequency measurement there was a main effect of condition (F(1,44) = 9.091, p = 0.004) but not for the duration measurement.

### Obedience behavior

Finally, we looked at the frequency and duration of the dogs' obedient behaviors like sitting and lying down. Again we conducted two 2 (condition)×2 (variant)×2 (intonation) repeated measures ANOVAs which revealed that there was no significant interaction between all three factors (duration: F(1,44) = 0.343, p = 0.0.561; frequency: F(1,44) = 1.086, p = 0.303). There were also no significant interactions between the factors intonation and variant (duration: F(1,44) = 0.446, p = 0.508; frequency: F(1,44) = 0.272, p = 0.605). There was also no significant interaction between the factors intonation and condition neither for the frequency nor for the duration measurement (duration: F(1,44) = 0.032, p<0.858; frequency: F(1,44) = 0.000, p = 1.000). Furthermore, there was a main effect of intonation for the duration measurement revealing that dogs showed longer obedience behavior in the imperative trials over the informative trials regardless of any other factor (duration: F(1,44) = 4.487, p = 0.040).

**Figure 3:**
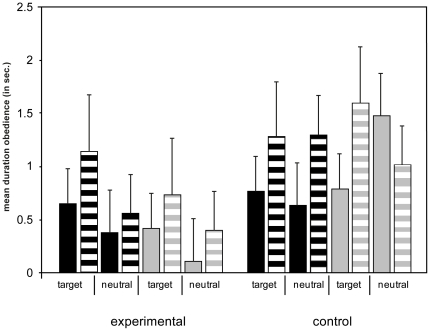
Obedience behavior. Mean duration (in seconds) of the obedience behavior (sit/laying down) in “target side” and “neutral side” in the experimental and the control condition, the context and no context trials and the informative and imperative trials (SE). Black bars represent the context trials; grey bars represent no context trials. Non-lined bars represent informative trials, lined bars represent imperative trials.

And again we conducted two 2 (condition)×2 (variant)×2 (intonation)×2 (halves) repeated measures ANOVAs to look at the dogs' location in the room while performing those behaviors and compared whether dogs performed the actions within the “target side” or the “neutral side”. Again there were no significant four-way interactions between all factors nor were there any significant three-way interactions between any factors. But there was a significant two-way interaction between the factors halves and condition for the frequency but not for the duration measurement (duration: F(1,44) = 2.701, p = 0.107; frequency: F(1,44) = 4.616, p = 0.037) revealing that dogs showed more obedience behavior in the target side over the neutral side but only in the experimental condition regardless of context or no context trials (see fig. 3). Additionally, there was a significant interaction between the factors halves and intonation for the frequency but not for the duration measurement (duration: F(1,44) = 1.436, p = 0.237; frequency: F(1,44) = 7.061, p = 0.011) revealing that dogs showed obedience behavior more often within the ‘target side’ than within the ‘neutral side’ but only when the experimenter used an imperative tone of voice and irrespective of condition (Figure 3). No other factor or their interactions were significant.

## Discussion

The current study demonstrates that dogs respond differently to human pointing with gaze alternation depending on contextual information in a communicative situation.

Specifically, dogs that had experienced a food-related foraging situation beforehand searched for longer when they saw a human pointing to an empty location on the ground as compared to those dogs that had not experienced such a context. Importantly, the dogs' behavior in the control condition (where they had the same experience but the experimenter did not point) demonstrates that indeed the pointing gesture was the initiator for the dogs to search in the experimental condition as they searched significantly less when there was no communication at all. This suggests that dogs expect to find a referent when they see the human point somewhere.

Interestingly, for the frequency measure of the dogs' searching indicates that the dogs searched more often in the context trials than in the no context trials – regardless of condition. We suggest that the frequency measure indicates that dogs checked briefly whether there is food around or not. The duration measurement, however, might be indicative of enduring and intentional search behavior. That is, when the experimenter used the pointing gesture the dogs searched for a longer time span than when the experimenter only vocalized.

Importantly, the dogs did not search randomly after seeing a pointing gesture. If they had experienced the human pointing for them, dogs searched for longer and more often in the area that the human was pointing to. In addition to that and more importantly, they searched longer in the direction the human was pointing to but only in those trials where they had experienced a food context prior to the communicative situation.

Interestingly, had dogs not experienced finding food beforehand, they ignored the pointing gesture during their search and did not prefer to search in the direction indicated.

This shows that dogs do not follow a pointing gesture irrespective of the context in which they receive it. Importantly, in the control condition, the dogs also searched to the same amount in both halves of the room.

These findings may also contradict a purely associative account of point-following as it has been suggested by some researchers [10]–[11]. If dogs had simply learned to associate the hand gesture with food we would expect them to search in the direction of the gesture no matter what context has been established previously. Instead, the dogs only seemed to expect to find something upon following the gesture when they had reason to do so. However, one may argue that something like conditional discrimination is underlying the dog's behavior in this situation. Future research will show whether this is the case.

Another finding of the current study is that the human's intonation has an effect on the behavior of the dogs. Dogs showed more frequent search behavior when they were addressed with a high-pitched, friendly tone of voice than an imperative, command-like tone of voice. This was only found when they had seen the human pointing for them compared to the control condition but regardless of whether there was a food context prior to this communicative situation or not. It could be that a high-pitched voice in combination with a pointing gesture rouses the dogs, which then triggers more activity, resulting in more search behavior in general. In contrast to the increased search activity in the informative trials, dogs sat or laid down in the direction of the pointing gesture more often when they were being addressed with an imperative command-like tone of voice.

The most likely explanation for this is that the imperative tone of voice is triggering obedient behaviors. Other work has also shown that the human's tone of voice can have an effect on dog's obedient behaviors [12], [13].

Thus, the results of the current study support the view that dogs do not follow the human pointing gesture ‘blindly’, but instead take contextual information into account.

Several studies have demonstrated that dogs follow pointing to objects like e.g., cups or containers containing a reward [1]–[2], [14] in a communicative situation. Studies investigating gaze-following in dogs have found that dogs will not follow a human's gaze to an empty space [2], [8]. But these studies investigated whether dogs would recognize the human gaze as a mental state of attention, and not what the dogs understand about a human's intentional communicative act towards a referent.

To gain information about the processes which underlie dogs' comprehension of human communicative acts one has to investigate dogs' responses to the communicative acts of humans, just as has been conducted in this study. To our knowledge this is the first study looking at dogs' comprehension of the pointing gesture while pointing to an empty space with no referent being present. Dogs showed a highly flexible use of the pointing gesture and their response depends on the context as well on the human's underlying communicative motive. Purely associative explanations do not account for their behaviour. Future studies will need to be conducted in order to ascertain if our findings about dogs' differential response to pointing in conditions with and without contextual information is evidence that dogs truly understand pointing as a referential communicative act.

## Supporting Information

Movie S1 **Example of the experimenter's voice.** Tone of the experimenter's voice in the imperative trials followed by tone of the experimenter's voice in the informative trials. Both examples show the experimental condition (with pointing gesture).
(MPG)
